# *Mycobacterium bovis* Infection, United Kingdom

**DOI:** 10.3201/eid1003.020819

**Published:** 2004-03

**Authors:** Robert M.M. Smith, Francis Drobniewski, Andrea Gibson, John D.E. Montague, Margaret N. Logan, David Hunt, Glyn Hewinson, Roland L. Salmon, Brian O’Neill

**Affiliations:** * National Public Health Service for Wales, Cardiff, United Kingdom; † Health Protection Agency, London, United Kingdom; ‡ Department for Environment, Food and Rural Affairs, Gloucestershire, United Kingdom; § Gloucester Hospitals National Health Service Trust (formerly Gloucester Public Health Laboratory), Gloucester, United Kingdom; ¶ Health Protection Agency (West Midlands) Regional Surveillance Unit, Birmingham, United Kingdom; # Veterinary Laboratories Agency, Weybridge, United Kingdom; ** Gloucestershire Health Protection Unit, Gloucester, United Kingdom

**Keywords:** Zoonoses, Communicable Disease Control, Cattle Diseases, Tuberculosis

## Abstract

We describe the first documented spillover of bovine tuberculosis from animals into the human population of the United Kingdom since the resurgence of the disease in cattle in the country. This finding suggests that there may be a small risk for transmission to humans, making continued vigilance particularly necessary.

In the past, *Mycobacterium bovis* was a major source of tuberculosis in humans through consumption of unpasteurized milk. Currently, tuberculosis as a result of *M. bovis* infection is comparatively rare, but it remains a cause for concern in persons at high risk, such as abattoir workers (*1*). *M. bovis* principally affects cattle, but it can cause disease in a range of wild and domesticated animals, for example, badgers, ferrets, cats, deer, and llamoids (*2*). In U.K. cattle, *M. bovis* infection is now primarily a pulmonary disease, and the main route of transmission is likely to be through aerosol dissemination. Currently, approximately 1% of human tuberculosis cases can be attributed to *M. bovis*; most of those are likely to follow reactivation (*3*) or to be recent infections contracted abroad. Tuberculosis caused by *M. bovis* in the young is usually a primary infection. We report two human cases in Gloucestershire. One of the cases may have resulted from intrafamilial spread.

## Case Studies

Two siblings—one currently residing, the other residing until recently, on their parents’ farm in Gloucestershire—were diagnosed with bovine tuberculosis in 1999. A brother and sister ages 20 and 17 years, respectively, they are thought to have the first cases of indigenously acquired bovine tuberculosis caused by *M. bovis* in persons <25 years of age, with no documented history of travel abroad, reported to the Public Health Laboratory Service Communicable Disease Surveillance Centre (CDSC) since 1990.

When seen by her physician in 1999, the 17-year-old girl had a 6-month history of cough, weight loss, and lethargy. Infection with acid and alcohol fast bacilli (AAFB) was confirmed by culture of bronchial washings. Her brother had an 18-month history of cough. He was subsequently found to be AAFB-smear positive with pulmonary cavitation (i.e., he had an infectious case). Sputum samples from both cases were cultured by the Gloucester Public Health Laboratory, and the cultures were identified as *M. bovis* by the Regional Centre for Mycobacteriology in Cardiff. All human *M. tuberculosis* isolates are subjected to biochemical analysis and pyrazinamide drug susceptibility testing for differentiation of *M. bovis*. *M. bovis* is therefore detected as part of the routine reference service.

Both siblings had lived on the same farm most of their lives. However, the sister had recently moved into her own place at the time of her diagnosis. Both patients had received *Mycobacterium bovis* BCG in secondary school. Both smoked. Neither had knowingly drunk unpasteurized milk. The girl had no cattle contact. Her brother had occasional cattle exposure: he would assist when stock were confined in a cattle crush^1^ for veterinary examination and restrained them by holding their nostrils. During this process, he could become covered in bovine mucus and saliva. He also reported contact with feral ferrets.

No disease has been reported in other family members or in social contacts. Results of screening of other family members (mother, father, and another sibling) were unremarkable. Their father had a grade 2 Heaf test result (and a previous history of BCG) (this is equivalent to a Mantoux response of induration of diameter 5 to 14 mm). Their mother had two grade 1 Heaf test results (Mantoux response of 0 to 4-mm induration) and no history of BCG. The other sibling (age 8) had a grade 1 and a grade 2 Heaf test result and no history of BCG. Heaf grades 0 and 1 or a Mantoux response of 0 to 4 mm induration are regarded as negative; those with a grade 2 reaction (or a Mantoux response of induration of diameter 5 to 14 mm following injection of 0.1 mL purified protein derivative 100 U/mL) are positive. Persons with a grade 2 response are hypersensitive to tuberculin protein and are not given BCG vaccination. A strongly positive reaction to tuberculin is demonstrated by a Heaf grade 3 or 4 or a Mantoux response with induration of at least 15-mm diameter.

The farm had previously held a maximum of 25 beef cattle, introduced around 1981. Tuberculosis (*M. bovis*) herd breakdowns^2^ had been recorded by the former Ministry of Agriculture, Fisheries and Food. Five cattle (of 7 slaughtered in a herd of 15) had bovine tuberculosis in 1993; they had caseous lymph node lesions and were culture positive. Another three (out of the herd of eight, which were all slaughtered) had similar lesions in 1997. All infected cattle showed lesions typical of *M. bovis* with confirmatory culture obtained; one had prescapular lymph nodes enlarged with caseous changes. The remaining animals were slaughtered as “direct contacts.” Tuberculosis breakdowns have been reported in neighboring herds, and the area supports a substantial badger population. After the 1993 cattle breakdowns, five badgers were trapped; four were positive for *M. bovis* on culture. Similarly, in 1997, a single trapped badger was culture positive.

## Conclusions

*M. bovis* from the cases and from cattle on the farm in 1997 were indistinguishable by a combination of restriction fragment length polymorphism (RFLP) analysis using the IS6110 element, spacer oligonucleotide (“spoligotyping”), and variable number tandem repeat (VNTR) analysis (*4**–**6*). RFLP analysis using the IS6110 insertion sequence represents the standard criterion for differentiating *M. tuberculosis,* but it is insufficiently discriminating for *M bovis* due to the paucity of IS6110 elements in the genome of this bacterium. Spoligotyping is based on the polymerase chain reaction (PCR) amplification of a polymorphic direct repeat (DR) locus in which the DR elements are interspersed with up to 43 spacer regions (Figure). The typing process relies on the presence or absence of spacers in the amplified DNA, which are detected by hybridization to a series of synthetic spacer oligonucleotides covalently linked to a filter. The presence of hybridized areas is shown by using a chemiluminescent reaction detected on film as a dark band; absence of spacers shows no binding. The sequence is then displayed as a binary bar code, which can be manipulated digitally. Similarly, VNTR analysis uses PCR to amplify a region in which there are tandem repeats at multiple loci. The result is a digital code describing the number of repeat units at each locus (Figure). The spoligotype profile obtained in these cases is one of the most common seen in bovine tuberculosis in the United Kingdom, and caution is needed before one can say unambiguously that strains have been transmitted. Nevertheless, the combination of typing methods, together with supportive epidemiology, provides evidence of exposure to a common source of infection.

**Figure:**
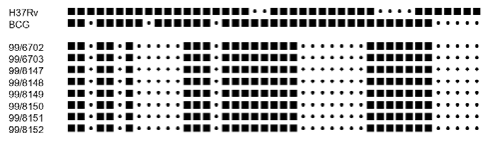
Spoligotyping profiles for human and cattle cases of bovine tuberculosis. H37Rv and BCG are control strains. 99/6702 and 99/6703 are the sister and brother, respectively; 99/8147–99/8152 are cattle isolates.

*M. bovis* was characteristically transmitted to humans by ingestion of infected milk. Thus, historically, human *M. bovis* lesions were primarily extrapulmonary or intestinal. Cattle infected with *M. bovis*, by contrast, usually have pulmonary infection, and shedding of *M. bovis* in respiratory secretions has been reported by several workers (*7*–*9*). It is suggested that a possible route of badger to cattle transmission is by inhalation of bacilli from grass contaminated with infected badger urine, feces, or sputum (*10*). Cattle preferentially graze edges of fields, and they may sometimes be forced to graze close to badger latrines and scent-marking areas at the edge of fields. Cattle-to-cattle transmission of *M. bovis* is also likely to be important. Work to date (*11*) indicates that particular tuberculosis spoligotypes are usually clustered in specific areas, implying that herd breakdowns are localized events originating from a relatively static reservoir. In many instances, cattle and badgers have been found to share similar spoligotypes (*11*), but further sampling of badgers, cattle, and other wildlife is required to identify which species can share the infection. Current Department for Environment Food and Rural Affairs research is aimed at establishing the epidemiology and pathogenesis of *M. bovis* and the possible pathways of interspecies transmission.

Agricultural workers may acquire the disease by inhaling cough spray from infected cattle. Typical pulmonary tuberculosis then develops, which is what we believe occurred here.

Despite a long history of cattle herd breakdowns on this farm, the family members were not screened until the human cases occurred. Early detection of the disease in the young man before it became infectious might have prevented transmission to his sister and avoided the need for chemoprophylaxis for her infant son. No guidelines were in force at the time. Those subsequently issued (*12*) advocate screening of human contacts of disease only where pulmonary or udder lesions are detected in cattle. Since the early 1980s, reports of cattle herd breakdowns have steadily risen, with a more dramatic increase since 1990. The Southwest of England, the West Midlands, and South and West Wales have had recent increases in disease in cattle, and this trend is extending northward to include Derbyshire, Staffordshire, and Shropshire. This incident represents the first documented probable spillover into the human population from animals since the disease’s resurgence in cattle, and it suggests there may be a small risk for transmission to humans, even when the bovine case is reported as closed,^3^ because of the presence of *M. bovis* in the cattle’s respiratory tract (*7*).

## Footnotes

1 A cattle crush is a small, often portable, cage used to restrain individual animals. Crushes are often used in the United Kingdom to hold animals for veterinary attention.

2 A tuberculosis breakdown is confirmed when a visible lesion, typical of tuberculosis, is seen by an official veterinary officer in the carcass of a tuberculosis reactor at postmortem or, if *M. bovis* is cultured from the set of lymph glands that are routinely collected at postmortem and sent to a state veterinary laboratory for culture.

3 “Closed” refers to a gastrointestinal tract case in which the lymph nodes had not caseated and there was no perceived contact with the outside of the body cavity, as there would be a with a known respiratory (open) infection involving the lungs.
